# Robust myoelectric pattern recognition methods for reducing users’ calibration burden: challenges and future

**DOI:** 10.3389/fbioe.2024.1329209

**Published:** 2024-01-22

**Authors:** Xiang Wang, Di Ao, Le Li

**Affiliations:** ^1^ Institute of Medical Research, Northwestern Polytechnical University, Xi’an, China; ^2^ Research and Development Institute of Northwestern Polytechnical University in Shenzhen, Shenzhen, China

**Keywords:** electromyography (EMG), HD-sEMG, myoelectric pattern recognition (MPR), robust myoelectric control, electrode shift, cross-subject, cross-scenario

## Abstract

Myoelectric pattern recognition (MPR) has evolved into a sophisticated technology widely employed in controlling myoelectric interface (MI) devices like prosthetic and orthotic robots. Current MIs not only enable multi-degree-of-freedom control of prosthetic limbs but also demonstrate substantial potential in consumer electronics. However, the non-stationary random characteristics of myoelectric signals poses challenges, leading to performance degradation in practical scenarios such as electrode shifting and switching new users. Conventional MIs often necessitate meticulous calibration, imposing a significant burden on users. To address user frustration during the calibration process, researchers have focused on identifying MPR methods that alleviate this burden. This article categorizes common scenarios that incur calibration burdens as based on data distribution shift and based on dynamic data categories. Then further investigated and summarized the popular robust MPR algorithms used to reduce the user’s calibration burden. We categorize these algorithms as based on data manipulate, feature manipulation and, model structure. And describes the scenarios to which each method is applicable and the conditions required for calibration. Finally, this review is concluded with the advantages of robust MPR and the remaining challenges and future opportunities.

## 1 Introduction

The surface electromyography (sEMG) signal is an electrophysiological signal that records muscle activity by placing electrodes on the surface of the skin (Scheme and Englehart, 2011). As it contains movement information and is non-invasive, it can be used to decode motor intent. Many researchers consider it ideal for controlling devices such as prosthetics and exoskeletons (Hudgins et al., 1993). Over the years, myoelectric pattern recognition (MPR) technology has been a breakthrough, enabling multi-degree-of-freedom prosthetic control (Scheme and Englehart, 2011), offering hope for the rehabilitation of people with disabilities. In recent years, MPR technology has found wider application in human-computer interaction and has even demonstrated potential in consumer electronic devices. For example, it is utilized in MI devices for virtual reality, gaming entertainment, and industrial control (Zhang et al., 2009; Taneichi and Toda, 2012; Khalaf et al., 2020). Although the performance of myoelectric interfaces (MIs) is satisfactory under laboratory conditions, there are usually a variety of practical challenges in real-lift applications (Parajuli et al., 2019; Stephanidis et al., 2019).

Real-life applications often present dynamic environments, where conditions change over time (Stephanidis et al., 2019; Fleming et al., 2021). However, traditional MIs are typically trained based on the assumption of data being identically and independently distributed (*i.i.d*) (Jiang and Farina, 2014). This assumption implies that the training and testing of the classifiers occur under the same conditions, including users, electrode positions, and command categories. Consequently, this lack of adaptability and variability can significantly impact performance of the system or render it unusable (Rodriguez-Tapia et al., 2020). Hence, traditional MIs require frequent calibration—a redundant, time-consuming, and labor-intensive process that imposes a substantial burden on users. These calibration burdens stand as a primary factor contributing to the abandonment of myoelectric interfaces (Jiang and Farina, 2014; Fleming et al., 2021).

**FIGURE 1:**
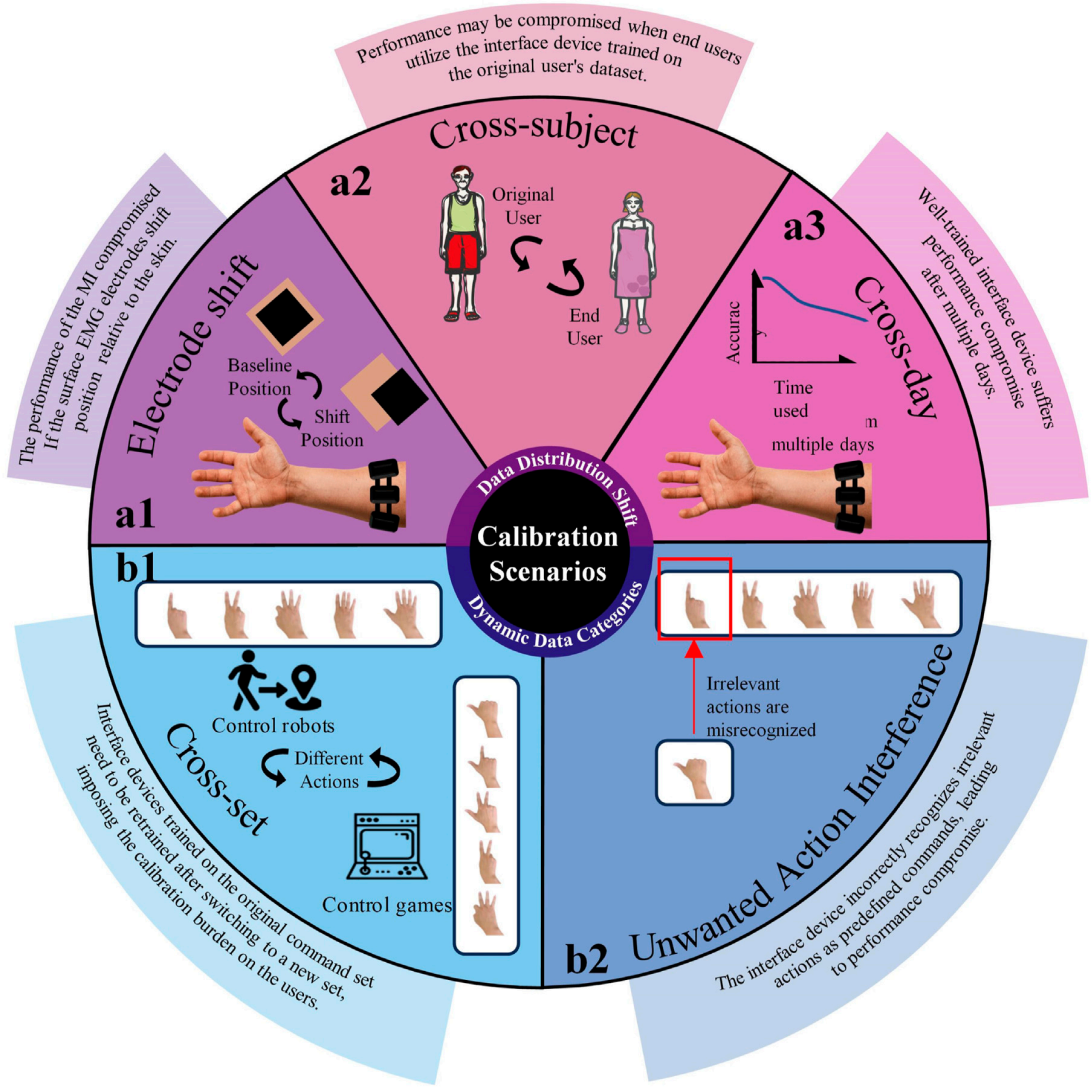
Illustration of the calibration scenarios required after two types of data shift have occurred. Data distribution shift includes electrode shift, cross-user, and cross-day (a1–a3). Dynamic data categories include cross-set and unwanted action interference (b1-b2).

Some of the common calibration burdens are, for example, that the electrode positions may shift due to sweaty skin or large movements of the user (Ameri et al., 2020); that the recognition accuracy is compromised by the user-dependent characteristics of the EMG signals after switching users (Phinyomark et al., 2021); that the recognition accuracy fluctuates over time due to changes in the environment or the user’s own physiological conditions (Jiang and Farina, 2014; Donati et al., 2023); that the MIs become disabled due to ineffective training after alternating commands used for control (Wang et al., 2023a); and that the user performs actions outside of the command set which are incorrectly recognized as being inside the command set during execution of the movement (Wu et al., 2021). These calibration burdens are essentially brought about by asymmetries in the distribution of data domains (Wang et al., 2021). For instance, the shift happens from the source domain to another target domain after the calibration burden has occurred. The shift is typically observed in the following application scenarios, which are divided into two types in the present article: data distribution shift and dynamic data categories (Figure 1).

## 2 Calibration burden scenarios

### 2.1 Data distribution shift

Data distribution shift occurs when there are changes in electrode position, user switching, or long-term wear, resulting in data within the source and target domains to no longer exhibit independent and identical distribution (*i.i.d)* (Zhang et al., 2022; Kou et al., 2023). These shifts in data distribution often coincide with electrode shifts, cross-user scenarios, and changes across different days.

#### 2.1.1 Electrode shift

Electrode shift can be caused by repositioning the device or by limb movement, which can lead to variations in the EMG signal. These variations can have an impact on the accurate recognition of motor intent by the MI (Wu et al., 2020). Electrode shift has been observed in both separated electrode and high-density electrode arrays (Isaković et al., 2022). In the case of 4-channel separated electrodes, a 1-cm shift increases misclassification by 15% (longitudinal shift) to 35% (lateral shift) (Yang et al., 2018). Similarly, with 10 × 10-channel HD-sEMG electrodes, a 7-mm shift leads to nearly 15% misclassification (right-distal) to 30% misclassification (left-proximal) (Wu et al., 2020). Electrode shift is considered almost unavoidable in MIs. Therefore, there is a need to improve the robustness of MIs to overcome the disturbances.

#### 2.1.2 Cross-subject

**FIGURE 2:**
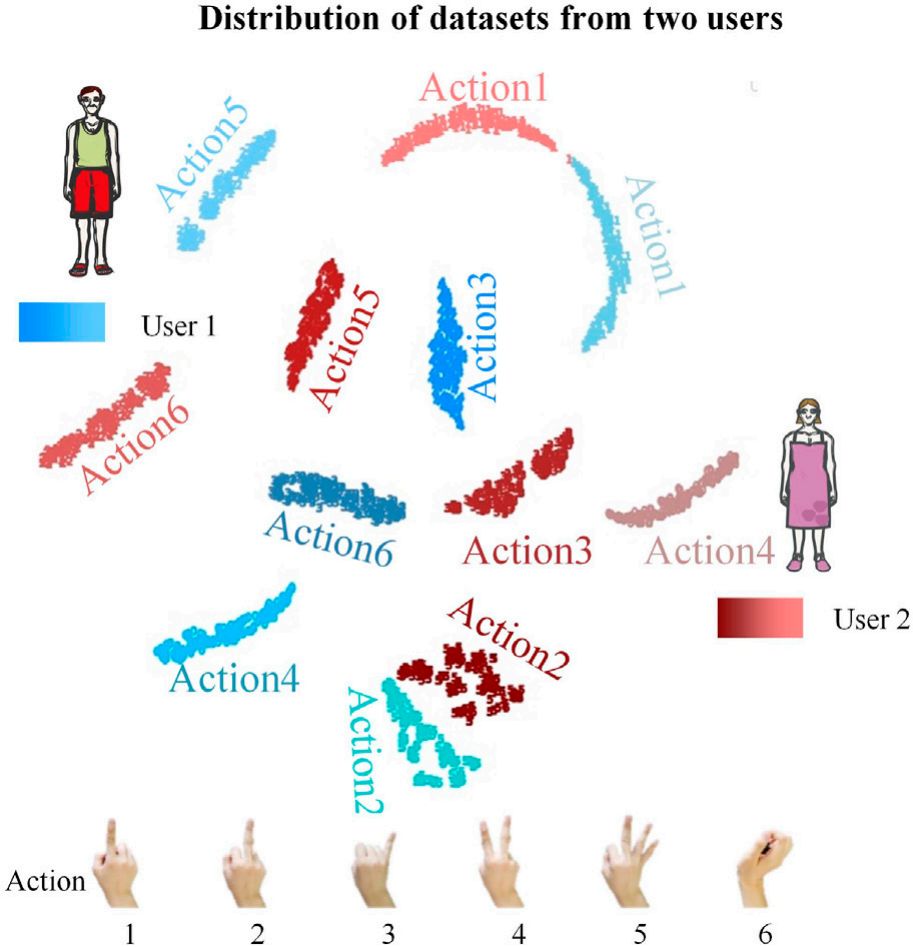
The t-SNE visualization shows the data distribution of the same six actions for two users. The blue data is from user 1 and the red data is from user 2, with different shades representing different actions. The difference in data distribution between two users is significant even when they perform the same actions.

When performing the same action or movement task, sEMG signals exhibit significant variations among users due to their non-stationary random characteristics and differences underlying MI (Stephanidis et al., 2019; Wu et al., 2019; Rodriguez-Tapia et al., 2020). Factors such as fat volume, number of muscle fibers, and skin impedance can impact sEMG measurements (Phinyomark et al., 2021; Zhang et al., 2022). Figure 2 illustrates the data distribution for 2 users performing 6 identical actions. Significant differences can be seen in the data distribution between different users even when performing the same action. For healthcare applications, where multiple users alternate in using public devices, frequent calibration is required (Jiang and Farina, 2014; Fleming et al., 2021). In the case of private prostheses, although they are be used by multiple users, differences in the distribution of pre-training data and end-user data exist, necessitating a long learning period for calibration during the initial use (Cote-Allard et al., 2020). Additionally, for MIs utilized in consumer electronics, seamlessly switching between multiple users is equally crucial (Wang et al., 2022; Wang et al., 2023b). Therefore, the ability to cope with differences in multiple users is important for commercial MIs.

#### 2.1.3 Cross-day

A state-of-the-art MI should exhibit stability and repeatability; however, in practical applications, the sEMG signal shows significant variations over time (J. Wu et al., 2019). These variations can be attributed to electrode shifts caused by device wear-off and physiological factors such as muscle fatigue and changes in body temperature (N. Jiang and Farina, 2014). Therefore, achieving a cross-day stable MI requires more comprehensive robustness, which also presents a greater challenge (Jiang et al., 2022a). Some studies (Phinyomark et al., 2013; Waris et al., 2018) suggest that training data collected over several days (days >5) can be effectively enhance recognition accuracy. However, this approach is impractical for real-world applications (Jiang and Farina, 2014). Hence, it becomes essential to enhance the cross-day robustness of the MI to reduce the calibration burden.

### 2.2 Dynamic data categories

Dynamic data categories are characterized by variations in the action categories between the source and target domains. These discrepancies may arise users requiring additional or alternative action commands, or from the presence of unwanted interfering actions in the target domain.

#### 2.2.1 Cross-set

Whether in consumer electronics or prosthetics, effectively switching between command sets for various application scenarios is crucial (Zhang et al., 2009; Kim et al., 2016). For instance, in rehabilitation training based on game interaction, referring to diagnostic results to establish a rehabilitation prescription (command set) is often necessary (Taneichi and Toda, 2012). However, the calibration process after switching sets can be excessively time-consuming and labor-intensive, particularly when recording data for new actions, which also requires a considerable amount of time (Wang et al., 2023a). Traditional MIs barely work when they cross-sets but have not been calibrated. Therefore, being able to quickly update the MIs’ control commands is necessary to improve the flexibility and is also a challenging calibration scenario for myoelectric interfaces.

#### 2.2.2 Unwanted pattern interference

The majority of MIs are trained using a fixed command set consisting of a limited number of actions (Rodriguez-Tapia et al., 2020). These MIs rely on predefined action commands. However, users should not restrict themselves to only a few specific actions, and many unintended interfering actions are often misidentified as trained actions by the MI. Such unwanted actions can not only lead to misclassification or even compromise the functionality of the entire system (Simão et al., 2019). This misidentification significantly impacts the accuracy of MPR (Wu et al., 2021).

## 3 Robust MPR methods

The researchers propose various solutions for the aforementioned calibration scenarios. Initially, calibration methods involved data re-collection and model retraining. Subsequently, updating the electrode configuration also became a calibration method. While these methods improved the robustness of MI, they still imposed a significant calibration burden on users due to their cumbersome operation. In recent years, MPR has achieved remarkable success in the field of MIs, and robust MPR methods show potential for alleviating the calibration burden. These robust MPR methods usually depend on strategies involving data manipulation, feature manipulation, and model structure. Table 1 provides a summary of representative studies on multiple methods, describing the specific calibration scenarios targeted by each method and the required conditions for calibration. In addition, Table 2 summarizes the advantages and shortcomings of these methods.

**TABLE 1:** Comparison of conditions required for different calibration methods.

MPR methods	Strategies		Calibration Scenarios	Labeled Calibration Data	Unlabeled Calibration Data	Re-training	Related Works
Data Manipulate	Data Augmentation Manipulates		Electrode Shift;	Not Necessary	Not Necessary	Not Necessary	Chamberland et al. (2023);
			Cross-day;				Jiang et al. (2022a);
			Cross-subject				Jiang et al., (2022b);
							Islam et al. (2022);
							Wu et al. (2020)
	Core Activation Zone Extraction		Electrode Shift	Not Necessary	Need	Not Necessary	Hu et al., (2021); Zhang et al., (2020)
Feature Manipulate	Feature Alignment		Cross-subject; Electrode Shift	Not Necessary	Need	Need	Wang et al., (2023b);
							Donati et al., (2023);
							Kou et al., (2023);
							Zhang et al., (2022);
							Fan et al. (2022);
							Li et al. (2023)
	Feature Metric		Novel Action Interference	Not Necessary	Not Necessary	Not Necessary	Jiang et al., (2022a);
							Wu et al., (2021);
							Li et al. (2023)
	Feature Optimization		Cross-day;	Not Necessary	Not Necessary	Not Necessary	Jiang et al., (2022b); Scheme and Englehart (2014)
Model Structure	Pretraining-finetuning		Electrode Shift; Cross-subject	Need	Not Necessary	Need	Chappell et al., (2022); Yang et al.,. 2018; Ameri et al., (2020)
	Adversarial Learning		Cross-subject; Cross-day	Not Necessary	Need	Need	Côté-Allard et al., (2020);
							Phinyomarkb et al., (2021);
							Cote-Allard et al. (2020)
	Meta-learning	Few-shot One-shot Zero-shot	Cross-set; Cross-subject; Electrode Shift	Need	Not Necessary	Not Necessary	Wang et al., (2023a); Phinyomark et al., (2013); Rahimian et al., (2021); Proroković et al., (2020)
	Multi-task Learning		Electrode Shift; Cross-subject	Need	Not necessary	Need	Kulwa et al., (2023);
							Zhang et al., (2023);
							He et al. (2020)

**TABLE 2:** Comparison of characteristics and shortcomings of several robust MPR methods.

MPR Methods	Strategies		Advantages	Shortcomings
Data Manipulate	Data Augmentation Manipulates		1. No need for calibration data. 2. Applicable to any model. 3. Executing this process imposes almost no burden on users.	Recognition accuracy improvement is not significant for scenarios involving category changes.
	Core Activation Zone Extraction			
Feature Manipulate	Feature Alignment		Can be employed to minimize the gap between domains within each distinct category.	1. Significant depreciation in recognition accuracy when there is insufficient unlabeled calibration data. 2. Recognition accuracy improvement is not significant for scenarios involving category changes.
	Feature Metric		Can be utilized to widen the gap between distinct categories.	
	Feature Optimization		1. No need for calibration data. 2. Applicable to any model. 3. Executing this process imposes almost no burden on users.	Recognition accuracy improvement is not significant for scenarios involving category changes.
Model Structure	Pretraining-finetuning		Higher recognition accuracy.	1. There is still a large burden of data collection and model retraining. 2. Recognition accuracy improvement is not significant for scenarios involving category changes.
	Adversarial Learning		No need to retrain the model.	1. There is still need burden of unlabeled data collection. 2. End-user applications with unsatisfactory recognition accuracy over time. 3. Recognition accuracy improvement is not significant for scenarios involving category changes.
	Meta-learning	Few-shot One-shot Zero-shot	1. Higher flexibility. 2. No need to retrain the model.	1. Few-shot and One-shot require labeled calibration data. 2. Zero-shot eliminates the need for calibration data but recognition accuracy is not ideal.
	Multi-task Learning		1. A model can be developed customized for each new scenario. 2. Higher recognition accuracy.	1. Model structure is complex and slow to respond. 2. Model structure needs to be updated after switching usage scenarios. 3. Insufficient flexibility.

### 3.1 Based on data manipulate

Data manipulation method plays a crucial role in enhancing the performance and generalization ability of MPR classifiers. There are two common strategies in this method: pre-processing manipulates strategy and core activation zone strategy.

#### 3.1.1 Data augmentation manipulates

Data augmentation is a straightforward and powerful technique solution that involves applying deformations to labeled training samples, generating extra training data while preserving the semantic meaning of the labels (Wu et al., 2020). One reason for this is that expanding the dataset enhances the model’s ability to generalize, which aligns with common understanding. Another reason is that specific data augmentation techniques enable the model to adapt to the data in the target domain. Common data augmentation operations include rotation, panning, random channel masking, adversarial generation, and more. These techniques have been extensively verified for their effectiveness in MPR. In a study by Wu et al. (Wu et al., 2020), the training set data was generated through simulating HD-sEMG images with fictitious shift positions, which effectively reduced the misclassification rate. The randomized channel masking technique proposed by Jiang et al. (Jiang et al., 2022b) is another effective strategy that generates new data with added perturbations based on the training data. This newly generated data becomes more complex and diverse, which benefits the enhancement of generalization ability in classifiers, particularly those focusing on cross-day scenarios. Lin et al. (Lin et al., 2023) proposed an adversarial-based perturbation data augmentation method that generates synthetic HD-sEMGs. These synthetic signals are utilized to train robust deep-learning models, enabling them to withstand interference from real HD-sEMG signals. There is also a data augmentation technique based on signal processing methods that generates synthetic data for increasing the data amount. Tsinganos et al. (Tsinganos et al., 2020) proposed a synthetic sEMG based on signal amplitude warping and wavelet decomposition techniques to add synthetic sEMG to the dataset, which improves the pattern recognition accuracy. Adjustment by electrode placement to improve the robustness of the model is another solution. Yang et al. (Yang et al., 2018) recommended placing all the electrodes in opposite directions during the training process and introducing the data after randomly switching the two channels into the training set to improve the robustness of MPR. The current robust MPR based on data augmentation is typically a straightforward additional strategy applied solely to the source domain data (Gu et al., 2022). It involves generating virtual data for the target domain, thereby enhancing the diversity of training samples, to improve the model’s generalization performance against perturbations. Data augmentation serves the purpose of increasing the volume of data, minimizing user data collection time, and enhancing model accuracy, ultimately aiming to alleviate the calibration burden.

#### 3.1.2 Core activation region extraction

Extracting core activation region is a commonly used data manipulation technique for electrode shifting. Previous research has suggested that the core activation region of human muscles remains relatively fixed and consistent for the same actions (Zhang et al., 2020; Hu et al., 2021). Consequently, the detrimental impact of electrode shifting on MPR performance can be alleviated by extracting the core activation region across various repetitive movements. Zhang et al. (Zhang et al., 2020) employed a deep neural network to detect and match muscle activation in HD-sEMG. They employed a partially overlapping region between the training and test images, serving as the core activation region. MPR was achieved by training solely on the core activation region. During testing, the system scans the entire array image of the test samples to locate and detect the core activation region Hu et al. (Hu et al., 2021) implemented a FastICA-based algorithm for extracting the core activation region before the MPR classifier. Subsequently, the training and test samples were converted into core activation region samples for MPR. Strategies based on the extraction of core activation regions aim to mitigate the impact of shifts in data distribution by preserving common information within a specific subset of the samples. This approach reduces the frequency of model calibration by minimizing the effect of data shifts to ease the calibration burden on the user.

### 3.2 Based on feature manipulate

Information extracted from EMG signals is represented as feature vectors. Feature extraction is considered a crucial step in MPR (Scheme and Englehart, 2014), as a well-chosen combination of features can minimize MPR errors (Hudgins et al., 1993). There are various common feature combinations, such as time-domain (TD) features proposed by Hudgins et al. (Hudgins et al., 1993). In recent years, extensive studies have been conducted to enhance the robustness of MPR through feature manipulation. Three commonly employed strategies include feature alignment, feature metrics, and feature optimization.

#### 3.2.1 Feature alignment

Feature alignment is a widely used strategy in feature manipulation that aims at minimizing the disparity between cross-domain data distributions. Zhang et al. (Zhang et al., 2022) employed the MMD distance function as a loss function to compute the distance between the source and target domains. Minimizing the distances between the source and target domains decreased the model’s gradient, achieving feature alignment. Kou et al. (Kou et al., 2023) developed a domain adaptive framework, known as second-order statistical distribution alignment (SSDA), that utilizes second-order covariance as a statistic and achieves alignment in both subspace and statistical distribution. Covariance characterizes the interrelationships between dimensions in a multidimensional space, offering insights into the overall situation. Leveraging these covariance characteristics improves the overall generalization of the system. Xue et al. (Xue et al., 2021) extracted inherent user-independent properties using canonical correlation analysis (CCA) and subsequently minimized inter-user distributional differences through the optimal transport (OT) framework. Moreover, many other robust MPR works are based on similar feature alignment strategies (Zhang et al., 2022; Wang et al., 2023a; Liu et al., 2023). Feature alignment involves computing the distance between the distributions of the source and target domains and subsequently minimizing this loss to achieve alignment between data domains. Aligning the data domain facilitates the adaptability of the originally trained model to the target domain, thereby reducing the need for model re-training.

#### 3.2.2 Feature metric

Feature metrics serve a different purpose compared to feature alignment, which not only reduces the distance between different domains but also aims to increase the data between different categories, thereby reducing the misclassification rate of the classifier. Therefore, this method can be used to reject unwanted actions. Wu et al. (L. Wu et al., 2021) employ a feature metric function to quantitatively measure the distribution distance between two samples, enabling them the identification and rejection of actions identify and reject unwanted actions that deviate significantly from the target actions. Chappell et al. (Chappell et al., 2022) employed the Wasserstein distance to compare the distribution of the input signal with a set of reference distributions. The was then classified based on the most similar distribution. Therefore, the feature metric strategy shares a similarity with the feature alignment approach as it involves calculating the distance between domains. However, the feature metric strategy aims not only to minimize the shift in domain distribution but also to directly classify or reject categories based on the computed distance. In certain cases, intentionally increasing the distance serves to alleviate classification challenges for the classifier. The need for frequent calibration of MIs can be alleviated by improving the performance of the classifier during cross-domain recognition.

#### 3.2.3 Feature optimization

Numerous studies propose novel feature combinations for various calibration scenarios, and these optimized combinations often demonstrate greater robustness compared to routine feature sets. Jiang et al. (Jiang et al., 2022a) utilized linear discriminant analysis to identify a set of high-performing feature combinations, highlighting the dependability of optimized features in classifying gestures across different days. Moreover, Scheme et al. (E. Scheme and Englehart, 2014) achieved significant improvements by substituting conventional time-domain features with innovative enhancements. The feature optimization strategy enables the model to acquire a generalized feature representation, enhancing its performance across domains. This results in improved cross-domain recognition accuracy and reduces the frequency of model calibrations. However, using this simple solution alone is still insufficient. Tkach et al. (Tkach, Huang, and Kuiken, 2010) suggest using feature optimization strategies as an adjunct in conjunction with effective classifier training strategies to further improve the robustness of MIs.

### 3.3 Based on model structure

With the rapid advancement of deep learning, numerous neural network models applied in computer vision and natural language processing have also influenced the field of MPR. However, designing a model structure for robust MPR is influenced by physiological factors, making not all models from computer-related fields applicable. The four primary strategies for achieving robust MPR through model structure include finetuning, adversarial methods, meta-learning, and multi-task learning.

#### 3.3.1 Pretraining-finetuning

Pretraining-finetuning refers to the process of improving the performance of a pre-trained model by adjusting specific parameters to accommodate changes in the data. This strategy typically necessitates access to labeled target domain data and involves retraining the model. Ameri et al. (Ameri et al., 2020) were the first to employ a depth model and finetuning approach to calibrate MI, with the goal of addressing the challenge of electrode shift. Despite its effectiveness, this method still required a substantial amount of data and calibration time. Subsequently, Chen et al. (Chen et al., 2021) introduced a novel model and finetuning framework that reduced the amount of calibration data and further alleviating the burden on the user. The pre-training-finetuning strategy involves modifying only specific parameters, thereby decreasing the time required for model retraining in comparison to a full re-training process.

#### 3.3.2 Adversarial learning

**FIGURE 3:**
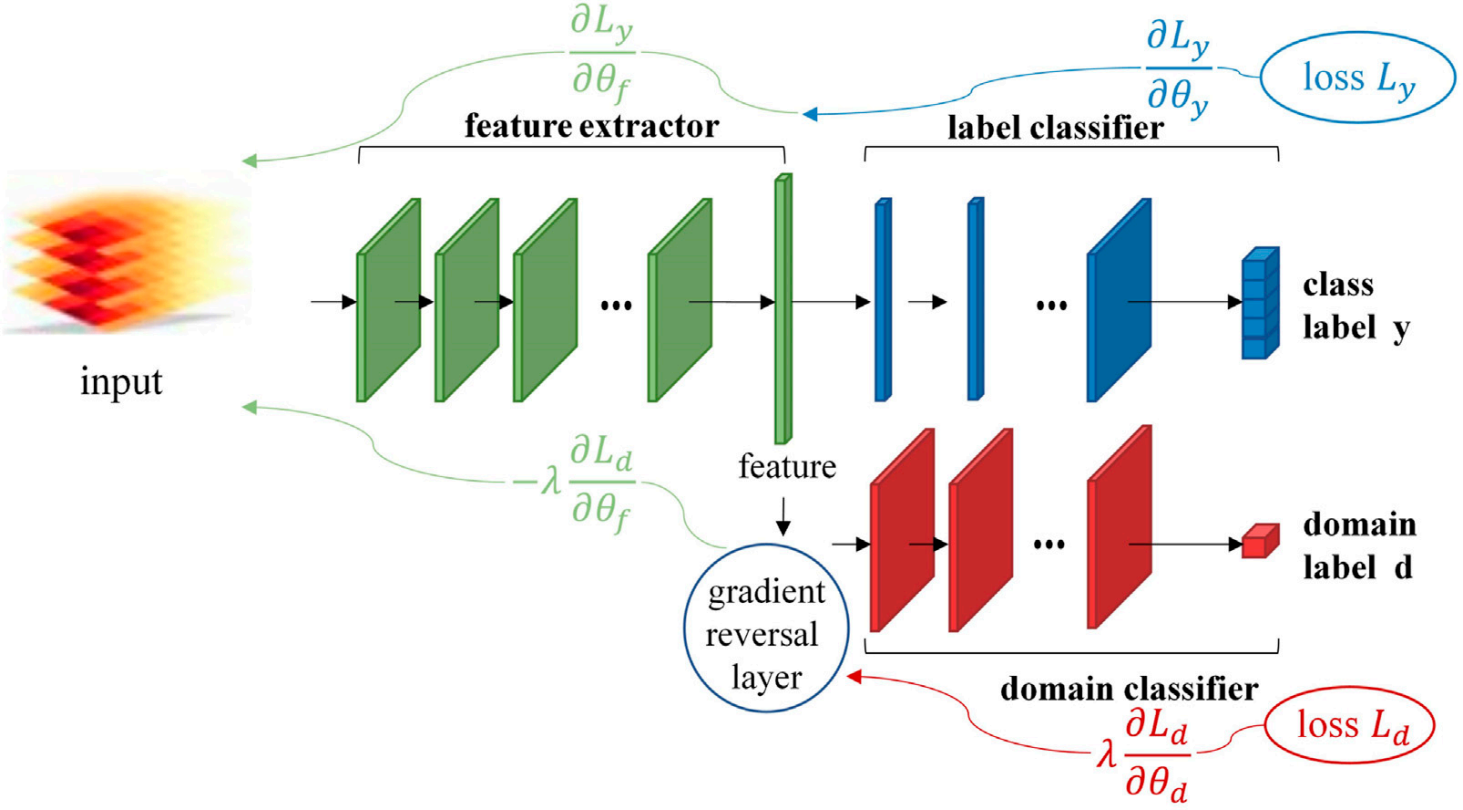
Illustration of the model architecture of DANN (Domain-Adversarial Neural Network). It contains green blocks (feature extractors), blue blocks (label classifiers) and red blocks (domain classifiers). The objective of model training is to minimize both label prediction loss (for source examples) and domain classification loss (for all samples). The gradient reversal layer is instrumental in ensuring that the feature distributions between two domains are made as similar as possible. This makes it exceedingly challenging for domain classifiers to discern differences between the domains. Such an operation is designed to generate domain-invariant representations that exhibit high similarity across various domains.

Although the finetuning strategy has demonstrated commendable performance in many cases, it still relies on labeled data for calibration. In contrast, the adversarial learning strategy provides independence from labeled calibration data. This approach is widely employed to acquire domain-invariant features. Ganin et al. (Ganin et al., 2016) were pioneers in introducing an adversarial neural network (DANN) for the field of computer vision (Figure 3). In this model, a discriminator is trained to distinguish between different domains, while a generator is trained to deceive the discriminator, facilitating the learning of domain-invariant feature representations. Cote et al. (Cote-Allard et al., 2020; Côté-Allard et al., 2021) proposed the adaptive domain adversarial neural network (ADANN) for MI, employing the adversarial concept to alleviate cross-domain data variations. The concept behind ADANN is to extract a universal feature representation from this multi-domain setting. Zhang et al. (Y. Zhang et al., 2023) proposed an improved conditional domain adversarial network (ICDAN). This model calculates the conditional domain adversarial network (CDAN) loss between source domain features and target domain features through a discriminator. The CDAN loss is employed to align features and categories. There are also many MPR algorithms based on adversarial learning which strategy is one of the most popular deep learning-based MPR algorithms (Y. Hu et al., 2019; Leite and Xiao, 2020; Choi et al., 2022). Adversarial learning strategies facilitate the unsupervised training of domain-invariant features between domains. This approach maximizes adaptation to both source and target domains, effectively reducing the need for model re-training and eliminating the requirement for labeled calibration data.

#### 3.3.3 Meta-learning

Meta-learning, referred to as learning-to-learn, has attracted considerable attention in recent years (Rahimian et al., 2021). Unlike traditional machine learning, models are trained with a focus on solving specific tasks. Meta-learning aims to enhance the generalization capabilities of the models, enabling them to learn rapidly and accurately with a limited number of samples and empirical data, even when faced with previously unseen tasks. Thus, meta-learning aligns better with the concept of natural interaction. It can address both the challenge of scarcity of training samples and facilitate swift command set switching. According to the amount of calibration data required, meta-learning is categorized into few-shot learning, one-shot learning, and zero-shot learning. Rahimian et al. (Rahimian et al., 2021) proposed the “few-shot learning hand gesture recognition” framework (FS-HGR) based on meta-learning. The framework combines temporal convolution and attention mechanisms. The objective is to rapidly calibrate MI using a limited number of calibration datasets (1-5 labeled samples per action category). Wang et al. (Wang et al., 2023b) improved the flexibility of MIs when crossing domains by leveraging a similarity function for one-shot learning, with only one labeled sample per action category. This approach utilizes a Siamese neural network to train a similarity function that evaluates the similarity between pairs of samples. In novel scenarios with newly introduced gesture categories and/or new users, rapid calibration of the MI can be achieved with just one sample per category. Al-Naser et al. (Al-Naser et al., 2018) proposed a framework based on zero-shot learning that utilized unlabeled samples. Unlike the idea of traditional frameworks that recognize fixed action categories, zero-shot learning involves setting action categories as predefined basic actions and related combinations of actions. Once an unknown action is recognized, it is automatically transformed into a predefined combination of basic actions, eliminating the need for calibration. Meta-learning demands a few calibration data, or even none (Zero-shot), for proficiently classifying unseen categories. It is especially well-suited for dynamic data categories tasks, facilitating rapid additions or substitutions in the command set, thereby enhancing the flexibility of MIs. This approach significantly reduces the calibration data requirement for users and eliminates the need for the model re-training process.

#### 3.3.4 Multi-task learning

Multi-task learning involves training a model to simultaneously handle multiple related tasks, rather than training separate models for each task as the routine strategy. The central concept of multi-task learning is to enhance the model’s generalization across all tasks by sharing knowledge and features. A related concept is multi-domain learning, which commonly applies the principles of multi-task learning. In multi-domain learning, the model is trained on data from various domains, to improve its adaptability to the data shifts occurring in these different domains. Consequently, this strategy enhances the model’s generalization performance, making multi-domain learning most suitable for addressing challenges related to data domain shifts. He et al. (He et al., 2020) introduced a position recognition framework that utilizes multi-domain learning to mitigate the effects of electrode shifts. In the training phase, data were gathered from both the initial position and potential shifted locations, and classifiers were trained using data from each specific location. In the testing phase, the user executed a specialized gesture designed to detect electrode shifts. This concise gesture contraction facilitated the selection of the optimal classifier, which was subsequently employed for subsequent myoelectric control. Kulwa et al. (Kulwa et al., 2023) proposed a dual-stage convolutional neural network (DS-CNN)-based model, where multiple convolutional neural network (CNN) models are trained in the first stage for shifts at different locations. The second stage then triggers the corresponding models based on the detected electrode shift locations to accurately decode the individual’s motor intention. Rahimian et al. (Rahimian et al., 2023) proposed dynamic multi-task learning, where a multi-task network can dynamically decide which parts of the network to activate based on the task and input samples, with the aim of exploiting the task and sample conditions to improve the weight-sharing flexibility of the multi-task network, and ultimately achieve better generalization among multiple tasks. Multi-task learning allows the model to perform well on multiple tasks at the same time by learning generalized representations for different domains, which in turn reduces the process of calibrating MIs.

## 4 Discussion

In summary, the robust MPR methods hold great potential for creating user-friendly MI devices. These methods boast various advantages, as we have outlined, the most significant being their capacity to alleviate the calibration burden on the user while enhancing the flexibility of the MIs.

### 4.1 Alleviating calibration burden

Robust MPR methods are implemented to alleviate the calibration burden for users. This burden usually consists of two parts: calibration data collection and model training time (Wang et al., 2023a). Minimizing the number of calibration samples and shortening the model training time can lead to a reduction. For MI devices, the model training time usually consists of two parts: pre-training time and re-training time. The pre-training time for robust MPR algorithms based on deep learning is indeed quite extended, sometimes reaching hourly durations. Nevertheless, this process can be computed in advance using a substantial amount of offline data, and the end-user is not inconvenienced by this phase. What can impact users’ calibration burden is typically the computational time for model re-training.

#### 4.1.1 Computational time for model re-training

The extended computation time of re-training significantly impacts the calibration burden on end-users. Prolonged waiting times, especially when switching scenarios, can adversely affect the overall user experience. However, recent advancements have resulted in novel models that substantially decrease or eliminate the necessity for re-training (Cote-Allard et al., 2020; Wang et al., 2023b). Some robust MPR methods, such as zero-shot learning, have been reported to completely eliminate the need for the model re-training process (Al-Naser et al., 2018). On the other hand, routine machine learning models such as KNN require only a millisecond level for retraining (Guo et al., 2003; Murugappan, 2011; Wang et al., 2023a). While certain robust MPR algorithms have demonstrated the elimination of model re-training time, routine machine learning methods generally maintain an acceptable level of re-training time. Therefore, computation time is not the primary contributor to the calibration burden on the end-user. Indeed, the significant breakthrough achieved by the robust MPR approach lies in the substantial reduction of calibration data.

#### 4.1.2 Re-collection process for calibration data

In traditional machine learning, four repetitions are performed. With each action category having a contraction time of 5 seconds, requiring over 3 minutes to collect data for just ten actions. In contrast, some robust MPR methods, such as zero-shot learning and data augmentation, do not require any calibration data (Rahimian et al., 2021; Wang et al., 2023b). Therefore, robust MPR methods offer substantial advantages in alleviating the calibration burden.

### 4.2 Enhancing flexibility

Flexibility plays a vital role in commercial MIs, as it enables them to swiftly adapt to the donning and doffing of prosthetics and wearable MIs. Moreover, these MIs must possess the capability to seamlessly switch between users, rapidly adjust the range of action commands, and handle various scenarios effectively (Jiang and Farina, 2014; Fleming et al., 2021). Research efforts have yielded targeted solutions for these challenges with some studies demonstrating the possibility of achieving plug-and-play functionality without the need for any calibration data (L. Wu et al., 2020; Al-Naser et al., 2018). These approaches provide substantial advantages over standard methods, which typically demand a large amount of labeled data collection and model updating during the switching process between command sets (Guo et al., 2003; Murugappan, 2011; Paul, Goyal, and Jaswal, 2017). In contrast, robust MPR methods greatly enhance the flexibility of MIs, making them more versatile and user-friendly.

## 5 Challenges and opportunities

Current MIs based on MPR often face limitations related to specific calibration burden scenarios, hindering their ability to switch seamlessly between different scenarios. As an example, an MI device that focuses on cross-users usually struggles to perform well cross-set (Rodriguez-Tapia et al., 2020). Moreover, specific MPR strategies, such as adversarial learning, require substantial amount of unlabeled data despite not necessitating labeled calibration data. However, the challenge lies in the fact that although unlabeled data can be gathered during the myoelectric interface’s usage, it cannot be directly utilized by end-users, particularly if it is not acquired sufficiently during the device’s initialization phase (Côté-Allard et al., 2020; Cote-Allard et al., 2020). This limitation impedes the direct usability of the MI in such situations. Although certain data and feature manipulation strategies have the potential to eliminate the need for calibration entirely, insufficient understanding of the target domain can result in decreased recognition accuracy (L. Wu et al., 2020). Additionally, it is crucial for the MPR algorithm to maintain stable recognition accuracy in both offline and online testing scenarios. Although Ameri et al. (Ameri et al., 2018) have demonstrated that there is no significant difference in pattern recognition accuracy between CNN models tested online and SVM models tested offline. However, for the same algorithm, there are also many studies reporting that the real-time accuracy is not as good as the offline accuracy (Zhang et al., 2020). Numerous robust MPR algorithms, including those reviewed in this article, have been evaluated primarily through offline testing. The potential oversight in focusing on real-time testing results may be attributed to the lack of standardized evaluation criteria (Hinson et al., 2023) or their limited deployment in the industry at present. Despite the promise shown by certain algorithms, like the DANN-based robust MPR algorithm implemented in a virtual reality system by Côté-Allard et al. (Côté-Allard et al., 2020), the authors categorize this testing as “dynamic dataset training” due to feedback solely from the visual camera. It is not a true online test. Hence, enabling effective online testing remains a challenge for robust MPR.

In the future, the overarching aim for MI is to achieve genuine “plug-and-play” functionality. This entails not only the capability for online applications but also the elimination of any calibration burden. Data-driven MIs relying solely on data face challenges in achieving satisfactory recognition accuracy due to the absence of target domain information. In recent years, integrating physiological knowledge, such as musculoskeletal information, has become a trend in MI research (Hu, et al., 2021; Li, Wang, et al., 2023). This approach, grounded in physiological modeling, ensures robustness in the face of EMG signal variations. Its effectiveness lies in the recognition that the formation of the EMG signal is influenced by a variety of factors, including skin condition, blood flow, fat composition, and temperature, among others, rather than being a simple linear combination of motor unit action potentials (MUAPs) (Farina, Stegeman, and Merletti, 2016; J. Wu et al., 2019). Musculoskeletal models encompass deterministic information, incorporating models like Hill-type muscle models and multilink arm dynamic models (Heine, Manal, and Buchanan, 2003; Pan, Crouch, and Huang, 2018). These models not only derive insights from EMG signals themselves but also map internal muscle force states and anticipated joint movements based on physiological features. Moreover, this approach excels in learning the user’s own intricate factors of variation. Hence, by employing the MPR method that integrates physiological knowledge, the model can gather more valuable information. It no longer treats EMG signals as a black box, directly mapping them to joint kinematics. This advancement enhances the performance and adaptability of the interface (Berman et al., 2023).
